# Description of two new *Lophocampa* Harris from the Dominican Republic (Arctiidae, Arctiinae)

**DOI:** 10.3897/zookeys.75.811

**Published:** 2011-01-12

**Authors:** Benoit Vincent

**Affiliations:** 5 place de l’Ermitage, 93 200 Saint Denis, France

**Keywords:** Phaegopterini, Arctiidae, Neotropics, new species, Dominican Republic, *Lophocampa*

## Abstract

Two new species of Lophocampa Harris are described from the Dominican Republic, Lophocampa lineata **sp. n.** based on two males, and Lophocampa albitegula **sp. n.** based on three females. The habitus and genitalia are illustrated. The following nomenclatural changes are also proposed: Lophocampa albiguttata Boisduval, 1870, **stat. rev.** and Lophocampa brunnea Vincent, **nom. n.**

## Introduction

The genus Lophocampa Harris includes 66 species and 10 subspecies in the most recent catalogue (Watson and Goodger 1986), with an additional 10 species subsequently described (Beutelspacher 1986, 1992; Vincent 2005a, 2005b, 2009), one raised from synonymy, and one omitted by Watson and Goodger (Vincent 2009). Two species-level names have also been synonymised (Vincent and Laguerre 2010). Six of the 10 new species were captured during a private entomological project in the Dominican Republic carried out by the author in 2004. This material was complemented by a mission carried out by Jean Haxaire and Odile Paquit in the mountains of this country in 2007, when two new Lophocampa were discovered. The purpose of this paper is to describe these two new species, and to compare them with closely related species. Two taxaare raised from synonymy as *bona* species, resulting in one junior homonym for which a replacement name is proposed.

## Methods and materials

Specimens were collected in the Dominican Republic by attraction to a mercury vapour light bulb, powered by a portable generator. Trapping was done throughout the night from 6:30 pm to 6:30 am. Specimens were injected with ammonia and stored in labelled paper envelopes. Dried specimens were subsequently relaxed in a humid container, mounted and spread. Genitalia were prepared using a hot KOH solution (10%). Illustrations were made using a camera attached to a Leica MZ16 stereomicroscope. The genitalia were stained with chlorazol-black to enhance membrane contrast with the cuticle, and were mounted on slides using Euparal. Terminology for the genitalic characters follows Klots (1970).

The treatment of the Arctiidae at the family level follows that of Mitchell et al. (2006) established on protein-coding nuclear genes. The tribal classification follows that of Jacobson and Weller (2002).

The “barcode” fragment of the mitochondrial cytochrome *c* oxidase subunit 1 (CO1) gene was used to compare molecular variation among taxa. Dried specimen legs, from the collection of the author, were sent to the University of Guelph (Ontario, Canada) and sequenced in the “All Leps Barcodes of Life Campaign” (BOLD). Extraction, amplification and sequencing protocols are described in Vaglia et al. (2008).

Repository abbreviations are as follows:

AMNHAmerican Museum of Natural History, New York, New York, USA

ANSPAcademy of Natural Sciences, Philadelphia, Pennsylvania, USA

BMNHThe Natural History Museum (formerly British Museum [Natural History]), London, UK

CMNHCarnegie Museum of Natural History, Pittsburgh, Pennsylvania, USA

MNHNMuséum National d’Histoire Naturelle, Paris, France

USNMNational Museum of Natural History (formerly United States National Museum), Washington, DC, USA

ZMHBMuseum für Naturkunde (formerly Zoologisches Museum, Humboldt Universität), Berlin, Germany

BVCPersonal collection of Benoit Vincent, Saint-Denis, France

## Systematics

### Lophocampa lineata

Vincent sp. n.

urn:lsid:zoobank.org:act:B379F5E6-D5BF-4897-816A-4561CEB03FA9

Figs 1 7, 9 

#### Type material.

***Holotype*** – ♂, Dominican Republic, Monseñor Nouel, Road El Blanco to Constanza pK [kilometer post] 10, Ebano Verde Scientific Reserve, 1360 m, 15-VIII-2007, 19°01.729'N, 70°30.988'W, J. Haxaire and O. Paquit *leg*. prep gen BV 355, Barcode ID ARCTB 641–08, Sample ID BEVI0551, Genbank # HQ682628. Deposited in MNHN. **Paratype.** 1 ♂, same data as holotype, Barcode ID ARCTB 873–09, Sample ID BEVI0768, Genbank # HQ682627 ; in [BVC].

#### Etymology.

The name refers to the two brown transverse lines crossing the forewing.

#### Diagnosis.

Lophocampa lineata Vincent, sp. n. is externally similar to Lophocampa propinqua Edwards 1884 (Fig. 2), but the two taxa can be separated by the forewing apex more rounded compared to Lophocampa propinqua, and the fringe not checkered at the vein terminals as in Lophocampa propinqua. The ratio of forewing length / width is 2.25 in Lophocampa lineata sp. n. (*n* = 2) and 2.48 in Lophocampa propinqua (*n* = 13). The male genitalia of Lophocampa lineata sp. n., compared to Lophocampa propinqua has a larger uncus without a spatulate apex and shorter valvae without strong spines on the basal protuberances (Figs 8 and 10). The CO1 barcode sequences of Lophocampa lineata sp. nov and Lophocampa propinqua differ by 7,62% - 7,79%.

**Figures 1–6.**
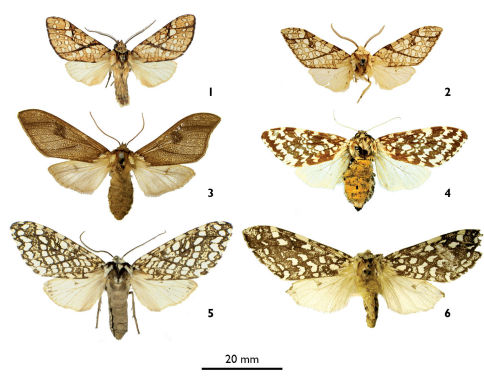
**1** Lophocampa lineata male holotype **2** Lophocampa propinqua male holotype **3** Lophocampa brunnea femaleholotype **4** Lophocampa alternata female holotype **5** Lophocampa albitegula female holotype **6** Lophocampa albiguttata female syntype potential.

#### Description.

##### Head.

Labial palpi curved upward, the third segment shorter than the first two. Yellow ventrally, brown dorsally. Frons yellow with a transverse large brown band. Vertex and scape yellow. Male antenna yellow with dark pectinations, longest rami 3.0 x longer than segment length.

##### Thorax.

Collar light yellow marked with two brown transverse lines located on each side of the median axis. Tegulaelight yellow with the internal side brown and with a light brownish spot centrally. Dorsal surface brown, with light yellow triangle centrally and the outer margins posteriorly light yellow. Ventral surface light yellow, very hairy. Prothorax brown. Meso- and metathorax light yellow. Legs yellow ringed with brown.

##### Forewing.

Length: 18 mm. Ground yellow with white spot bands as follows: basal band with one white spot highlighted with brown. Postbasal, antemedian, median, postmedian and subterminal bands formed by rounded white spots intercalating between veins. Postbasal band broken The postmedian band contains a large spot between veins M1 and M2 which is out of alignment with the rest. Subterminal band does not reach the anal angle, with the last spot reduced and triangular. The wing is crossed by two narrow brown transverse lines, the longest one is wide at the costa then narrows following vein CuA2. The shorter line follows vein M1. Fringe brown.

##### Hindwing.

White, semi-translucent; anal margin more densely scaled with yellow.

##### Abdomen.

Light yellow both dorsally and ventrally.

##### Male genitalia.

Tegumen triangular, large basally. Uncus elongated and rectilinear, slightly enlarged basally, bearing lateral setae except on the apical third; apex truncated. Valvae symmetrical, elongated, reaching the middle of the uncus, bearing a small, rounded protuberance near the apex. Basally, on the dorsal face, presence of two elongated, thin and symmetrical protuberances reaching the base of the uncus. Juxta reduced, trapezoidal with a small median notch. Vinculum slender. Penis straight, caecum penis reduced. Vesica large consisting of three smaller elongated lobes, lacking spines.

##### Female genitalia.

Unknown.

#### Biology and distribution.

It is reasonable to think that Lophocampa lineata sp. n. is a species restricted to middle elevations of the central cordillera in the Dominican Republic. Previous exploration in this area in April and May of 2004 failed to find this taxon. The habitat is montane cloud forest. Early stages and foodplants are unknown.

#### Remarks.

Based on a single male from Mexico, Edwards described Lophocampa propinqua as a variation of Lophocampa caryae Harris,1841. While examining the holotype in the AMNH collection, the specimen labelled *propinqua* from Jalapa, Mexico was examined. It has a white label with an unjustified lectotype designation by Allan Watson dated from 1967. The taxon *propinqua* was placed by Watson and Goodger (1986: 23) as a *bona* species in the genus Lophocampa Harris. The different forewing pattern, in particular the interrupted medial band, the narrower uncus and the longer valvae of the male genitalia justify this placement as a species distinct from the North American Lophocampa caryae.

Lophocampa caryae form *montana* Gaede, 1928 (Fig. 3) was described from Guatemala. In the catalogue of Watson and Goodger (1986: 23), this form is associated with Lophocampa propinqua. After consultation of the ZMHB collection where the type of *montana* Gaede is housed, it is evident that the habitus of *montana* is totally different from Lophocampa caryae, Lophocampa propinqua and in fact all Lophocampa species. Thus the taxon *montana* must be raised to species rank. However, Lophocampa montana (Gaede, 1928), stat. n. then becomes a junior secondary homonym of Lophocampa montana (Schaus, 1911), described from volcan Poás in Costa Rica. Consequently, I propose the replacement name Lophocampa brunnea Vincent, nom. n.

The nomenclatural changes proposed here are summarized as follows:

### Lophocampa brunnea

Vincent nom. n.

Lophocampa montana  (Gaede, 1928), stat. n., junior secondary homonym of Lophocampa montana (Schaus, 1911)

### Lophocampa albitegula

Vincent sp. n.

urn:lsid:zoobank.org:act:7651F821-F591-46F8-88E0-D57107FFF38A

Figs 5 11 

#### Type material.

***Holotype*** – ♀, Dominican Republic, Pedernales, track from los Arroyos to El Aguacate pK 5,2, 1990m, 11-VIII-2007, 18°16.269'N, 71°43.496'W, J. Haxaire and O. Paquit leg. prep gen BV 386, Barcode ID ARCTB 638–08, Sample ID BEVI0548, HQ682631. Deposited in [MNHN]. **Paratypes**. 2 ♀, same data as holotype, Barcode ID ARCTB 648–08, Sample ID BEVI0558, HQ682630, prep gen BV 369, Barcode ID ARCTB 639–08, Sample ID BEVI0549, HQ682629 ; in [BVC].

#### Etymology.

The name reflects the white tegulae of the species.

#### Diagnosis.

This species is superficially most similar to Lophocampa albiguttata (Boisduval, 1870) (Fig. 6).It has a dark ground colour on the forewings, a subterminal band formed by irregular and flattened spots, a postmedian band formed by spots rounded at the costa then similar to those of the subterminal band. The legs are ringed brown and white. Female genitalia have posterior apophysis shorter than the anterior (longer than the anterior apophysis in Lophocampa albiguttata) and a smaller bursa(Figs 11 and 12).

**Figures 7–12.**
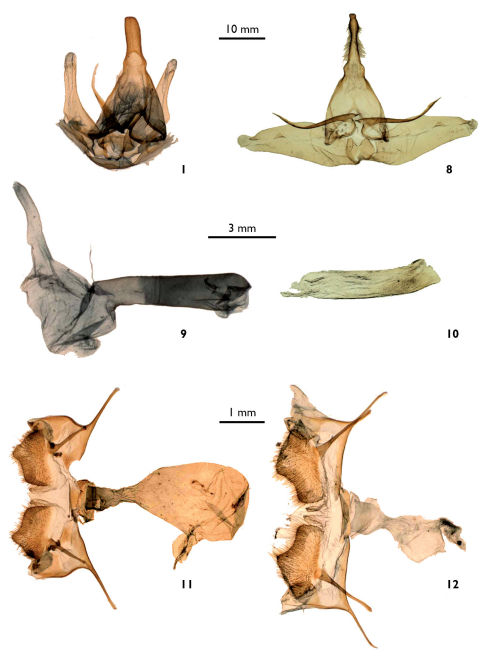
**7–8.** Dorsal view of male genitalia. **7** Lophocampa lineata male holotype (dissection # BV 355) **8** Lophocampa propinqua male holotype (A. Watson dissection # 1246). **9–10.** Lateral view of penis. **9** Lophocampa lineata male holotype **10** Lophocampa propinqua male holotype. **11–12.** Female genitalia, ventral view. **11** Lophocampa albitegula female holotype (dissection # BV 386) **12** Lophocampa albigutata female (dissection # BV 388), (Mexico).

#### Description.

##### Head.

Labial palpi dark, the third segment largely shorter than the two first. Frons dark, vertex white. Scape whitish. Antenna bipectinate, dark except the two first articles which are ochre.

##### Thorax.

Collar white except a wide median dark spot on the anterior side. Tegulae white, hairy, with the internal side dark and the center marked with a wide dark spot. Dorsal side dark with a central white spot and the median axis whitish on the posterior side. Legs dark with some whitish spots.

##### Forewing.

Length: 25 mmGround colour light beige, largely speckled with brown and ornamented with bands of white rounded spots underlined with brown.Basal band formed by an unique spot on the costa. Postbasal, antemedian, median, postmedian and subterminal bands formed by an alignment of white spots underlined with brown and intercalating between veins. Postbasal band broken. Antemedian formed by two white spots and two brown spots. Median band formed by two spots on the reniform spot. Postmedain almost straight and complete. Terminal band formed by irregular white spotssometimes fused with the sinuous subterminal spots.

##### Hindwing.

White, semi-translucent; anal margin more densely scaled with brown scales. The fringe is checkered white and brownish from apex to vein CuA2.

##### Abdomen.

Abdomen greyish.

##### Female genitalia.

Pseudopapillae analeswholly fused. Papillae anales trapezoidandstrongly setose. Anterior apophysis straight, 1 mm in length.Posterior apophysis with a long basis, 1,2 mm. Ductus bursaenarrow, non-sclerotized, reduced and wrinkled at the insertion with the corpus bursae. Corpus bursae large, ovoid, smooth without sigma.In its median area, insertion of a large ductus seminalis.

##### Male genitalia.

Unknown.

#### Biology and distribution.

It is reasonable to think that Lophocampa albitegula sp. n. is a species restricted to high elevations of the Sierra de Bahoruco in the Dominican Republic. It is probable that the new taxon could be present in the Sierra de Neiba. Previous exploration in this two area in April and May 2004 failed to find this taxon. The habitat is montane cloud forest. Early stages and foodplants are unknown.

#### Remarks.

Lophocampa albiguttata (Boisduval, 1870) is treated as a synonym of Lophocampa alternata (Grote, 1867) by Watson and Goodger (1986: 23). A comparison of the type material shows a clear difference in these two taxa, in particular the forewing pattern (fig. 4 and 6). Lophocampa albiguttata (Boisduval, 1870)р stat. rev. is therefore raised from synonymy. In the original description, Boisduval does not indicate the number of syntypes, based on specimens from Honduras. A female specimen from the Boisduval collection, conserved in the BMNH, bears a label “Oaxaca”, [Mexico] and a red “TYPE” label. This specimen from Oaxaca may be a syntype of Lophocampa albiguttata, although the species was described from Honduras. A locality error in the original description or labelling error could explain this contradiction. As there is some doubt about the status of this supposed type, the designation of a lectotype or neotype is currently not possible. It is also possible that a syntype is stored in another collection. Finally, no specimens from Honduras have been located to serve as potential neotype. Unfortunately, it was not possible to sequence CO1 on a recent specimen of this rare taxon and to compare it with Lophocampa albitegula sp. n.

Lophocampa alternata was described and illustrated based on a single female specimen (“Number 743, Gundlach’s MS. Catalogue” Grote 1867: 319) from Cuba, and the holotype was in the ANSP. Jason Weintraub, collection manager of this Institution, provided pictures of a female specimen labelled “TYPE n° 7695 by A.R. Grote” and “HOLOTYPE ♀ by A[llan] W[atson] 1967”. This specimen not currently in the ANSP. It may have been transferred to CMNH by mistake, during a major exchange of specimens between ANSP and CMNH in the mid-1960s (J. Weintraub, pers. comm.). The presence of this specimen, and other type specimens described by Grote from Cuba, is not yet confirmed by the CMNH’s curator.

## Supplementary Material

XML Treatment for Lophocampa lineata

XML Treatment for Lophocampa brunnea

XML Treatment for Lophocampa albitegula
